# Associations between purine metabolites and monoamine neurotransmitters in first-episode psychosis

**DOI:** 10.3389/fncel.2013.00090

**Published:** 2013-06-11

**Authors:** Jeffrey K. Yao, George G. Dougherty, Ravinder D. Reddy, Wayne R. Matson, Rima Kaddurah-Daouk, Matcheri S. Keshavan

**Affiliations:** ^1^Medical Research Service, VA Pittsburgh Healthcare SystemPittsburgh, PA, USA; ^2^Department of Psychiatry, University of Pittsburgh School of MedicinePittsburgh, PA, USA; ^3^Department of Pharmaceutical Sciences, University of Pittsburgh School of PharmacyPittsburgh, PA, USA; ^4^Medical Research Service, Bedford VA Medical CenterBedford, MA, USA; ^5^Department of Psychiatry, Duke University Medical CenterDurham, NC, USA; ^6^Department of Psychiatry, Beth Israel Deaconess Medical Center and Harvard UniversityBoston, MA, USA

**Keywords:** schizophrenia, first-episode psychosis, neuroleptic-naïve, oxidative stress, purine catabolism, monoamine neurotransmitters

## Abstract

Schizophrenia (SZ) is a biochemically complex disorder characterized by widespread defects in multiple metabolic pathways whose dynamic interactions, until recently, have been difficult to examine. Rather, evidence for these alterations has been collected piecemeal, limiting the potential to inform our understanding of the interactions amongst relevant biochemical pathways. We herein review perturbations in purine and neurotransmitter metabolism observed in early SZ using a metabolomic approach. Purine catabolism is an underappreciated, but important component of the homeostatic response of mitochondria to oxidant stress. We have observed a homeostatic imbalance of purine catabolism in first-episode neuroleptic-naïve patients with SZ (FENNS). Precursor and product relationships within purine pathways are tightly correlated. Although some of these correlations persist across disease or medication status, others appear to be lost among FENNS suggesting that steady formation of the antioxidant uric acid (UA) via purine catabolism is altered early in the course of illness. As is the case for within-pathway correlations, there are also significant cross-pathway correlations between respective purine and tryptophan (TRP) pathway metabolites. By contrast, purine metabolites show significant cross-pathway correlation only with tyrosine, and not with its metabolites. Furthermore, several purine metabolites (UA, guanosine, or xanthine) are each significantly correlated with 5-hydroxyindoleacetic acid (5-HIAA) in healthy controls, but not in FENNS at baseline or 4-week after antipsychotic treatment. Taken together, the above findings suggest that purine catabolism strongly associates with the TRP pathways leading to serotonin (5-hydroxytryptamine, 5-HT) and kynurenine metabolites. The lack of a significant correlation between purine metabolites and 5-HIAA, suggests alterations in key 5-HT pathways that may both be modified by and contribute to oxidative stress via purine catabolism in FENNS.

## INTRODUCTION

**FIGURE 1:**
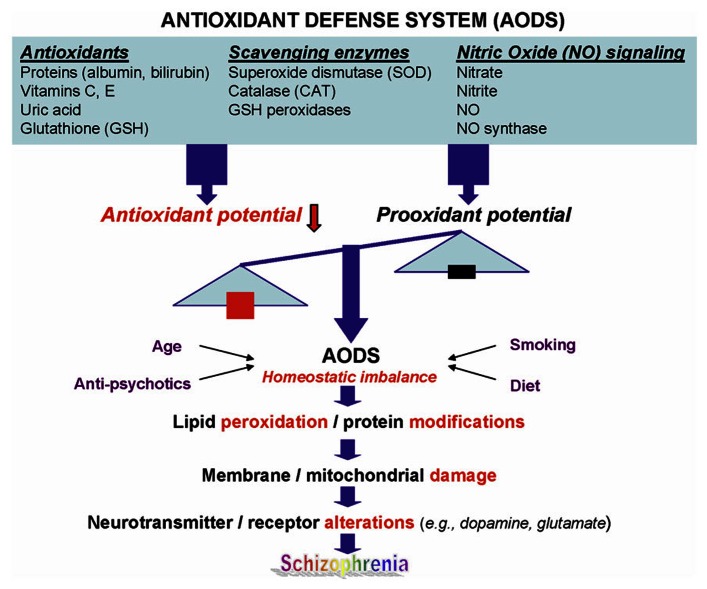
**A schematic diagram linking antioxidant defense system, membrane phospholipids, and neurotransmission to pathophysiology in schizophrenia.** Reprinted by permission from Yao and Keshavan (2011).

Schizophrenia (SZ) is a common and highly disabling mental disorder without a clearly identified pathophysiology. A number of putative mechanisms have been proposed to explain the etiopathogenesis and illness presentation of SZ including abnormal neuronal development, impaired neurotransmission, viral infections *in utero*, autoimmune dysfunction, and many others. Extensive, albeit fragmentary, findings from neurochemical and neuroendocrine studies of SZ (Javitt and Laruelle, 2006) have not provided conclusive evidence for any specific etiologic theory of SZ, perhaps due to etiopathogenetic heterogeneity (Tandon et al., 2009). However, there exists a point of convergence for many of these theoretical models, one that occurs at the level of the neuronal membrane, which is the site of neurotransmitter receptors, ion channels, signal transduction, and drug effects. Membrane deficits, specifically free radical-mediated, can significantly alter a broad range of membrane functions. There is abundant evidence that alterations in key neurotransmitters can both be modified by and contribute to oxidative stress and membrane dysfunction (**Figure 1**), suggesting a link among oxidative stress, membrane dysfunction, and multi-neurotransmitter pathologies in SZ (Yao and Keshavan, 2011).

## METABOLOMIC INVESTIGATION

Schizophrenia is a heterogeneous disease with various abnormal metabolites involving multiple biochemical pathways. Therefore, to identify candidate pathological process(es) that account for the constellation of clinical and biological features in SZ, it is necessary to simultaneously evaluate multiple metabolites in a network of interacting biochemical pathways. The development of high-resolution multidimensional separation techniques such as high-pressure liquid chromatography coupled with a 16-channel coulometric multi-electrode array system (HPLC–CMEAS), can lead to revolutionary changes in our understanding at the molecular level (Matson et al., 1984; Kristal et al., 1998; Yao and Cheng, 2004; Rozen et al., 2005; Kaddurah-Daouk et al., 2008). The resolving power of these methods is superior to one-dimensional approaches, enabling the comprehensive metabolic analyses particularly in the targeted biochemical pathways. The HPLC–CMEAS allows quantitative assays of hundreds to thousands of low molecular-weight metabolites, in turn permitting identification of biomarkers and metabolic maps associated with disease processes. The data collected from HPLC–CMEAS system reflect fingerprinting of the disorder or state/trait-related markers, which will greatly improve the predictive diagnostics for phenotypes that directly involve in the oxidative stress. More significantly, these comprehensive analyses that generate metabolic profiles represent not only biomarkers for disease but also metabolic maps that can be used to identify specific genes responsible for disease. Such metabolic maps provide a different perspective to biomedical research in further understanding the effects of therapeutic, nutritional, toxicological, and environmental interventions.

## ANTIOXIDANT DEFENSE SYSTEM

### GLUTATHIONE REDOX COUPLING AND NITRIC OXIDE SIGNALING

Free radicals are unstable atoms or molecules with odd (unpaired) electron(s) that can start a toxic chain reaction on important cellular components such as DNA, or the cell membrane. Biological systems have evolved complex protective strategies against free radical toxicity. Under physiological conditions the potential for free radical-mediated damage is kept in check by the antioxidant defense system (AODS), comprising a series of enzymatic and non-enzymatic components. These enzymes act cooperatively at different sites in the free radical pathways. A dynamic state is kept in check during the redox coupling under normal conditions (Yao et al., 2006). By contrast, lack of such correlations in brains of patients with SZ point to a disturbance of redox coupling mechanisms in the AODS, possibly resulting from a decreased level of glutathione (GSH) as well as age-related decreases of oxidized GSH and GSH reductase activities. Taken together, our previous data showing altered membrane dynamics and AODS enzyme activities, and findings from other investigators (Ranjekar et al., 2003; Othmen et al., 2008; Virit et al., 2009; Matsuzawa and Hashimoto, 2011) are consistent with the notion of free radical-mediated neurotoxicity in SZ (Yao et al., 2001).

There are multiple pathways to the production of excess free radical generation and subsequent oxidative stress. One such pathway is the formation of peroxynitrite by a reaction of nitric oxide (NO) and superoxide radical. In human brain, NO is metabolized primarily in the form of nitrate. A significantly increased level of NO was found in brains with SZ than those of normal and non-schizophrenic psychiatric controls (Yao et al., 2004a). Because the reaction of NO with free thiols competes with the same substrate (e.g., GSH), the excessive NO formation may further lead to significant depletion of GSH in SZ.

### PURINE CATABOLISM

**FIGURE 2:**
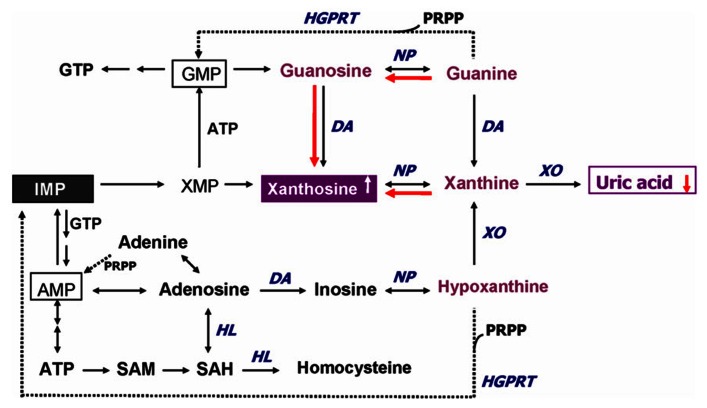
**Altered purine catabolism in first-episode neuroleptic-naïve patients with schizophrenia.** Red arrows indicate shifts toward an increase of xanthosine and a decrease of uric acid productions in FENNS patients at baseline. Reactions shown with dotted lines represent the “salvage pathways,” which purine bases can be reutilized resulting in considerably energy saving for the cell. ADP, adenosine diphosphate; AMP, adenosine monophosphate; ATP, adenosine triphosphate; DA, deaminase; GMP, guanosine monophosphate; GTP, guanosine triphosphate; HGPRT, hypoxanthine-guanine phosphoribosyltransferase; HL, hydrolase; IMP, inosine monophosphate; NP, nucleoside phosphorylase; PRPP, 5-phosphoribosyl pyrophosphate; SAH, *S*-adenosylhomocysteine; SAM, S-adenosylmethionine; XMP, xanthosine monophosphate; XO, xanthine oxidase. Reprinted by permission from Yao et al. (2010b).

In addition to GSH redox coupling mechanism and NO signaling, purine catabolism (**Figure 2**) may be a previously unappreciated component of the homeostatic response of mitochondria to oxidant stress and may play a critical role in slowing progressive mitochondrial dysfunction in certain disease states (Kristal et al., 1999). Mitochondria process most of the cellular oxygen to provide energy that drives almost all metabolic processes, and also are the site of significant free radical production. About 3% of all oxygen consumed is converted to superoxide, and subsequently to hydrogen peroxide (Floyd, 1996). Thus there is an enormous and continuous free-radical burden. Antioxidant systems keep this in check. When the equilibrium between pro-oxidant and antioxidant systems are disturbed in favor of the former, mitochondrial damage can occur. Mitochondrial membranes, similar to neuronal membranes, are vulnerable to lipid peroxidation. Any impairment in mitochondrial oxidative phosphorylation can lead to a broad range of cellular disturbances, including altered neurotransmission, increased DNA damage (Bogdanov et al., 2000; Schulz et al., 2000) and decreased DNA repair, and finally cell death. Cytochrome *c* oxidase is a key enzyme in the mitochondrial electron transport chain. Decreased activity of this enzyme has been reported in the frontal cortex and caudate nucleus of schizophrenic patients. Several lines of evidence suggest decreased oxidative metabolism in some brain areas in SZ (Yao et al., 2004a; Yao et al., 2006), and may be explained in part by mitochondrial dysfunction.

An early study by Kristal et al. (1999) indicated that purine catabolism may contribute to mitochondrial antioxidant defense by producing uric acid (UA). Failure to maintain elevated xanthine (Xan) and UA occurred contemporaneously with progressive mitochondrial dysfunction. Thus, purine catabolism appears to be a homeostatic response of mitochondria to oxidant stress and may protect against progressive mitochondrial dysfunction in certain disease states (Kristal et al., 1999).

During the *de novo* synthesis of purine nucleotides, many reactions require a great deal of energy utilizing the hydrolysis of adenosine triphosphate (ATP). To provide “energy saving” for the cell, the purine bases can be reutilized via “salvage pathways” (Cory, 1982) by converting adenine, guanine (G), or hypoxanthine (Hx) to adenosine monophosphate (AMP), guanosine monophosphate (GMP), or inosine monophosphate (IMP), respectively (shown dotted arrow in **Figure 2**). The unsalvaged Hx is then converted to Xan, which is further converted to UA by Xan oxidase. In man, UA is the final product of purine catabolism (Linden and Rosin, 2006), which has been implicated as a risk factor and cause of numerous pathological conditions (see below).

### DUAL ROLES OF URIC ACID IN AODS

Contrary to the traditional understanding as a metabolically inert and waste compound without any physiological significance, UA is a natural antioxidant contributing to approximately 60% of the free radical scavenging activity in human blood (Ames et al., 1981). Past studies have demonstrated that UA and inosine (precursor of UA) may be beneficial in the treatment of oxidative stress-related neurodegenerative diseases (Hooper et al., 2000; Spitsin et al., 2001; Scott et al., 2002; Liu et al., 2006; Du et al., 2007).

**FIGURE 3:**
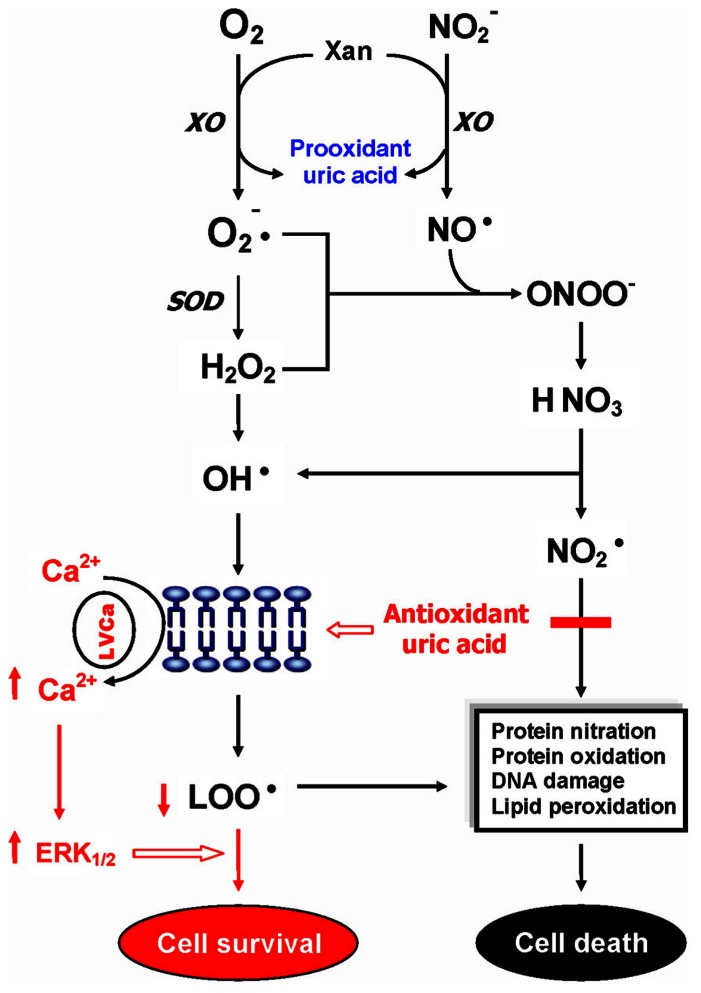
**Dual role of uric acid in the antioxidant defense system.** Uric acid can neutralize peroxynitrite and hydroxyl radicals to inhibit protein nitration and lipid peroxidation, respectively. At increased levels, however, uric acid may be considered as a marker of oxidative stress due to accumulation of reactive oxygen species. CAT, catalase; GSH, glutathione; ERK_1/2_, extracellular signal-regulated kinases½, LOO^•^, lipid peroxyl radical; LVCa, L-type voltage-gated calcium channel; NO, nitric oxide; ${NO}_{2}^{•}$, nitrogen dioxide radical; ${NO}_{2}^{-}$, nitrite; $O_{2}^{-}$, superoxide anion; ONOO^-^, peroxynitrite; OH^•^, hydroxyl radical; SOD, superoxide dismutase; Xan, xanthine; XO, xanthine oxidase. Reprinted by permission from Yao et al. (2010b).

Uric acid is a selective antioxidant (**Figure 3**) that removes superoxide by preventing the degradation of superoxide dismutase and subsequently inhibits its reaction with NO to form peroxynitrite (van der Veen et al., 1997). Moreover, UA can neutralize peroxynitrite (Keller et al., 1998) and hydroxyl radicals (Davies et al., 1986) to inhibit protein nitration (Pacher et al., 2007) and lipid peroxidation (Muraoka and Miura, 2003), respectively. Recent investigations further indicated that UA may operate as a protective factor mediated through astroglia for dopaminergic neurons from glutamate toxicity (de Lau et al., 2005; Du et al., 2007). Moreover, UA prevents the propagation of oxidative stress from the extracellular to the intracellular milieu by preserving the integrity of the plasma membrane at the lipid–aqueous interface boundary (Guerreiro et al., 2009). High K^+^-induced depolarization amplifies neuroprotection provided by UA through a mechanism involving Ca^2^ elevation and extracellular signal-regulated kinases½ (ERK_1/2_) activation (**Figure 3**). Thus, decreased plasma UA levels may reflect decreased ability of the body to prevent superoxide and peroxynitrite from acting on cellular components and damaging the cell (Kutzing and Firestein, 2007). Previously, we have demonstrated significant decreases of plasma UA levels in either first-episode neuroleptic-naïve patients with SZ (FENNS) patients (Reddy et al., 2003) or clinically stable patients with chronic SZ (Yao et al., 1998). Similarly, low levels of UA have been linked to a variety of neurodegenerative diseases including Alzheimer’s disease, multiple sclerosis, optic neuritis, and Parkinson’s disease (Church and Ward, 1994; Toncev et al., 2002; Knapp et al., 2004; de Lau et al., 2005; Kim et al., 2006; Bogdanov et al., 2008; Ascherio et al., 2009).

On the other hand, at increased levels, UA is considered as a marker of oxidative stress (Becker, 1993; Strazzullo and Puig, 2007) due to accumulation of reactive oxygen species (Hayden and Tyagi, 2004). Abnormally high levels of UA have been related to cardiovascular disease, gout, hypertension, and renal disease (Jossa et al., 1994; Freedman et al., 1995; Kang et al., 2002; Choi et al., 2005; Bos et al., 2006). Although some studies have indicated that UA may play a role in the development or progression of such diseases (Saito et al., 1978; Jossa et al., 1994; Waring et al., 2000; Kang et al., 2002; Bos et al., 2006), it remains unclear whether an increased UA contributes to the cause or simply a consequence of these pathologic conditions (Kutzing and Firestein, 2007).

In addition, an altered purine catabolism has also been demonstrated in subjects with cocaine addition (Patkar et al., 2009) or with opioid dependence (Mannelli et al., 2009), although plasma UA levels remained unchanged. It is not clear whether such changes in purine metabolites without affecting plasma UA levels would eventually lead to oxidative damage in substance abusers. Nevertheless, taken together, UA may serve as either antioxidant or pro-oxidant in the AODS as illustrated in **Figure 3**.

## HOMEOSTATIC IMBALANCE OF PURINE CATABOLISM

Using a targeted electrochemistry based metabolomics (HPLC–CMEAS) platform, we compared metabolic signatures consisting of six plasma purine metabolites simultaneously between FENNS (*n* = 25) and healthy controls (HC, *n* = 30). We also compared these metabolites between FENNS at baseline (FENNS-BL) and 4 weeks (FENNS-4w) after antipsychotic treatment (Yao et al., 2010b). Significantly higher levels of xanthosine (Xant) and lower levels of G were seen in both patient groups compared to HC subjects. Moreover, the ratios of G/guanosine (Gr), UA/Gr, and UA/Xant were significantly lower, whereas the ratio of Xant/G was significantly higher in FENNS-BL patients than in HC subjects (**Table 1**). Such changes remained in these same patients after 4 weeks of treatment (FENNS-4w) with the exception that the ratio of UA/Gr was completely normalized. During purine catabolism, both conversions from Gr to G and from Xant to Xan are reversible. Decreased ratios of product to precursor suggested a shift favorable to the Xant production resulting in decreased UA levels in the FENNS (**Figure 2**). More importantly, such an imbalance in purine catabolism is observed independent of treatment since patients were neuroleptic-nazïve at entry into the study.

**Table 1:** Comparisons of ratios of product to precursor in purine pathway.

Ratios	HC	FENNS-BL	FENNS-4w	*p*		
				HC vs BL^*^**	HC vs 4w^*^	BL vs 4w^†^
G/Gr	0.89 ± 0.61^§^	0.37 ± 0.30	0.48 ± 0.72	0.0004^¶^	0.0009	0.8949
Xan/G	46.33 ± 85.46	81.92 ± 98.86	66.68 ± 50.91	0.0211	0.0015	0.7112
UA/Gr	7371 ± 4325	4152 ± 2193	7047 ± 5556	0.0015	0.4967	0.0025
UA/G	11998 ± 11525	16529 ± 14751	23771 ± 14948	0.0614	<0.0001	0.0236
UA/Xant	5073 ± 4845	1298 ± 972	2184 ± 4310	0.0021	0.0067	0.5782
Xant/G	10.48 ± 15.58	42.02 ± 75.08	31.35 ± 27.93	0.0009	0.0001	0.2752

* Wilcoxon rank sum test.

† Wilcoxon signed rank sum test.

§ Data obtained from Yao et al. (2010b).

¶ Significance with *p* < 0.0033 after the Bonferroni correction.

*G, guanine; Gr, guanosine; Xan, xanthine; UA, uric acid; Xant, xanthosine*.

In addition, within the purines’ pathway, all three groups had significant correlations between G and UA, and Xan and Hx. By contrast, correlations of UA with each of Xan and Hx, and correlation of Xan with Gr were all quite significant for the HC group but not for the FENNS group before or after treatment. Thus, there are tightly correlated precursor and product relationships within purine pathways; although some of these correlations persist across disease or medication status, others appear to be lost among FENNS patients. Taken together, the potential for steady formation of antioxidant UA from purine catabolism is altered early in the course of illness (Yao et al., 2010b).

## CROSS-PATHWAY CORRELATIONS BETWEEN PURINE METABOLITES AND MONOAMINE NEUROTRANSMITTERS

The purinergic neurotransmission hypothesis was originally proposed in 1972 (Burnstock, 1972). Although ATP is widely recognized as an intracellular energy source for carrying out many biochemical reactions, it is also considered as a co-transmitter with glutamate, noradrenaline, acetylcholine, dopamine, and gamma-aminobutyric acid (GABA) in both central and peripheral nervous systems (Burnstock, 2007, 2009). Following the stimulation (e.g., electrical excitation) of brain, the adenine nucleosides that are stored in vesicles in nerve varicosities are released (Pull and McIlwain, 1972; Sulakhe and Phillis, 1975) by exocytosis to act on postjunctional receptors for ATP on smooth muscle. ATP is broken down by ATPases and 5′-nucleotidase to adenosine, which is taken up by varicosities to be resynthesized and reincorporated into vesicles. Adenosine is further broken down extracellularly by adenosine deaminase to inosine and Hx (**Figure 2**) and then removed by circulation (Burnstock, 1972).

In the study of normal behavior, purinergic signaling has been linked to learning and memory, sleep and arousal, locomotor activity and exploration, feeding behavior, and mood and motivation (Burnstock et al., 2011). On the other hand, a disordered purinergic signaling has been implicated in a variety of neurodegenerative diseases (Alzheimer’s, Parkinson’s, and Huntington’s disease, multiple sclerosis, and amyotrophic lateral sclerosis) as well as neuropsychiatric diseases (SZ and mood disorders). Previously, a conspicuous relationship was observed between purine and monoamine metabolite concentrations in cerebrospinal fluid (CSF) during depressive illness suggesting the presence of a parallel purinergic and monoaminergic activation in the brain (Niklasson et al., 1983).

To test whether plasma purine and monoamine metabolite concentrations were correlated in SZ, we studied previously published measurements (Yao et al., 2010a, b) of six purine metabolites (Hx, Xan, Xant, G, Gr, UA) for which concomitant measurements of 14 monoamine metabolites, tryptophan (TRP), serotonin (5-hydroxytryptamine, 5-HT), 5-hydroxyindoleacetic acid (5-HIAA), tryptamine (TRPA), melatonin (MEL), kynurenine (KYN), 3-hydroxykynurenine (3-OHKY), tryptophol (TPOL), tyrosine (TYR), L-3,4-dihydroxyphenylalanine (L-DOPA), Normetanephrine (NMET), homovanillic acid (HVA), 3-methoxy-4-hydroxyphenylglycol (MHPG), and vanillylmandelic acid (VMA), were also available from HC (*n* = 30) and FENNS-BL (*n* = 25) and FENNS-4w (*n* = 25). Using Q–Q plots and a univariate correlation test (Johnson and Wichern, 1998), we found these data not to be approximately normal nor consistently transformable to approximate normality for all three datasets (HC, BL, 4w). Kendall’s tau values and the *p*-values for rejection of H_0_: tau = 0, were thus computed for all pairs consisting of one purine metabolite and one monoamine metabolite within each of the three datasets. Correction of alpha for multiple tests (252) was done by the Bonferroni procedure.

The Kendall’s tau analysis found positive correlations that were significantly different from 0 in the HC group, for cross-pathway purine and monoamine metabolite pairs (**Table 2**) as follows: (1) for UA with TRP, 5-HIAA, MEL, KYN, and TYR; (2) for G with TRP, TYR, and possibly (trend) with MEL and KYN; (3) for GR with TYR; and (4) for Xan with TYR, and possibly (trend) with 5-HIAA. Many of these same correlations were also significant or possibly significant for the BL and 4w groups, with the following notable exceptions. The correlations between each of UA and Xan with 5-HIAA were much weaker and far from significance for BL and 4w patients, suggesting possible group differences among HC, BL, and 4w. Formal testing for equality of correlations among these groups, the next step, will require larger group numbers than are available with the present dataset.

**Table 2:** Across pathway correlations between 6 purine and 14 monoamine metabolites by the Kendall’s tau method.

Metabolites	Kendall’s tau rank correlations					
	HC (*n* = 30)		FENNS-BL (*n* = 25)		FENNS-4w (*n* = 25)	
I	II	tau	*p*	tau	*p*	tau	*p*
**Significant correlations among all three groups**
UA	TRP	0.6598	<0.0001	0.7122	<0.0001	0.6400	<0.0001
UA	MEL	0.5034	0.0001	0.5800	<0.0001	0.5400	0.00017
UA	KYN	0.6184	<0.0001	0.6800	<0.0001	0.6333	<0.0001
UA	TYR	0.7287	<0.0001	0.7200	<0.0001	0.6467	<0.0001
G	TRP	0.5034	0.0001	0.5843	<0.0001	0.5667	<0.0001
G	TYR	0.5816	<0.0001	0.5333	0.0002	0.5733	<0.0001
G	MEL	0.4667	0.0003	0.5267	0.0002	0.5600	<0.0001
**Significant correlations present only in HC and FENNS-BL but not FENNS-4w**
Gr	TYR	0.5681	<0.0001	0.6118	<0.0001	0.3022	0.0516
G	KYN	0.4805	0.0002	0.5200	0.0003	0.4933	0.0006
**Significant correlations present only in HC but not FENNS groups**
UA	5-HIAA	0.5310	<0.0001	0.1733	0.2336	0.3667	0.0109
Xan	5-HIAA	0.4759	0.0002	0.1733	0.2336	0.0133	0.9441
Xan	TYR	0.5264	<0.0001	0.4000	0.0054	0.4133	0.0041
**Significant correlations present only in FENNS-BL but not in HC and FENNS-4w**
UA	TRPA	0.3563	0.0060	0.6333	<0.0001	0.4267	0.0030
Gr	TRP	0.4613	0.0006	0.6440	<0.0001	0.3255	0.0359
Gr	MEL	0.4127	0.0021	0.6118	<0.0001	0.2480	0.1112
Gr	KYN	0.4127	0.0021	0.6256	<0.0001	0.3952	0.0107
Gr	3-OHKY	0.3885	0.0038	0.6403	<0.0001	0.3649	0.0187
**Significant correlations present only in FENNS groups but not in HC**
UA	3-OHKY	0.3977	0.0022	0.7114	<0.0001	0.5710	<0.0001

*Data were obtained from Yao et al. (2010a,b).*

*Significance with *p* < 0.000197 after the Bonferroni correction.*

*HC, healthy control subjects; FENNS, first-episode neuroleptic-naïve patients with schizophrenia; BL, baseline; 4w, 4-week after antipsychotic treatment; UA, uric acid; G, guanine; Gr, guanosine; Xan, xanthine; TRP, tryptophan; MEL, melatonin; KYN, kynurenine; TYR, tyrosine; 5-HIAA, 5-hydroxyindoleacetic acid; TRPA, tryptamine; 3-OHKY, 3-hydroxykynurenine.*

To summa rize, in HC, the purine and TRP pathways show extensive cross-correlations (all positive) among their respective member metabolites, whereas the TYR pathway shows significant cross-correlation with purines *only via tyrosine*. These relationships are generally seen for the BL and 4w groups as well. It may be that there are general dietary (precursor amino acids and purines are both high in many foods), hydration, hepatic, or other influences that affect purines and indoleamines and TYR similarly. However, the correlation of 5-HIAA with UA and Xan appear to be much weaker in the BL and 4w groups. We have already observed that BL patients have weaker correlations within the TRP pathway, e.g., 5-HIAA with TRP (tau = 0.09 BL, tau = 0.69 HC), which may occur when dietary associations are overcome by other rate-limiting pathway controls based on physiological needs for serotonin neurotransmitter (Yao et al., 2010a). The 4w group appears to have very little association between 5-HIAA and Xan, perhaps due to treatment with atypical neuroleptic drugs, which block serotonin 5-HT_2_, as well as dopamine D_2_, receptors, bringing more variables to influence the 5-HIAA metabolic product of 5-HT. The positive correlations in human CSF of Xan and several monoamines including 5-HIAA have been noted earlier (Niklasson et al., 1983) and between UA and 5-HIAA (Degrell and Nagy, 1990). It is notable that correlations are maintained between UA or G and metabolites in other branches of the TRP pathway (MEL, KYN) which are not involved in serotonin neurotransmission, for HC and patient groups.

## PURINERGIC SIGNALING, CLINICAL IMPROVEMENT, AND NEUROLOGICAL DEFICITS

**FIGURE 4:**
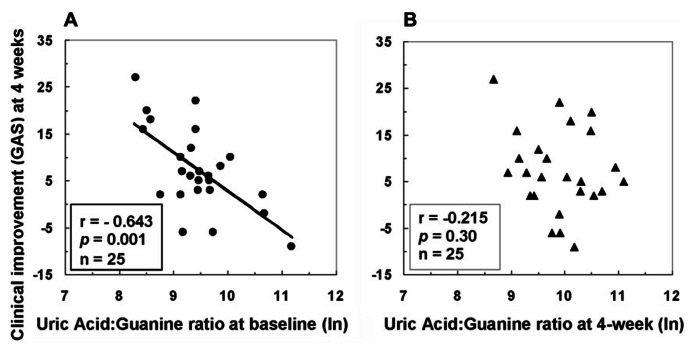
** Associations between clinical improvement at 4 weeks and ratio of uric acid to guanine in first-episode neuroleptic-naïve patients with schizophrenia at baseline **(A)** or at 4-week **(B)** after antipsychotic treatment.** GAS, Global Assessment Scale; ln, natural logarithm. Reprinted by permission from Yao et al. (2012).

Associations between purine metabolites and clinical and neurological symptoms were examined before and after 4w antipsychotic treatment (Yao et al., 2012). A lower initial proportion of product (UA) to precursor (guanine) measured at baseline was associated with *greater* improvement in clinical functioning 1 month later (**Figure 4**). Improvement in clinical functioning was associated with initial levels of UA and G in the FENNS patients. The initial severity of clinical dysfunction may thus be important to this relationship. As a group, the average level of clinical functioning reflected impairment at both time points, with mean values (<40) falling within the range typically observed for former inpatients likely to be readmitted to hospital (Endicott et al., 1976). Descriptively, degree of clinical improvement achieved by the patient group in the above study (Yao et al., 2012) represented an increase from “*Unable to function in almost all areas…*” at baseline to “*Major impairment in several areas…*” 1 month later (Global Assessment Scale or GAS anchor points). It may be appropriate, therefore, to qualify interpretation of findings based on this degree of severity.

Neurological abnormalities are a core feature of SZ even at the time of their first episode of psychosis without antipsychotic drug treatment (Rubin et al., 1994; Gupta et al., 1995; Keshavan et al., 2003; Mohr et al., 2003; Sanders et al., 2004). Moreover, neurological signs are correlated with clinical symptoms in unmedicated patients (Sanders et al., 2000). Significant heritability, or familial influence, has also been reported for several aspects of neurologic-related responding (Sanders et al., 2006), which suggest that neurological deficits may represent a biological marker of SZ risk. Recently, we have shown that purine metabolites were also linked to neurological and cognitive symptoms in the FENNS patients (Yao et al., 2012). Firstly, motor neurological signs (Buchanan and Heinrichs, 1989) recorded at baseline were associated with initial or baseline level of the ratio of Xant to Gr, indicating the higher a patient’s initial or baseline ratio of Xan to Gr, the greater his or her motor neurological signs was before initiating treatment with antipsychotic medications. Secondly, sensory-integrative neurological signs were predicted by baseline level of UA, which suggests that lower levels of UA were associated with *greater* impairment in sensory processing tasks. The above findings thus suggest an association between optimal levels of purine byproducts and dynamics in clinical symptoms and adjustment, as well as in the integrity of sensory and motor processing.

## PURINERGIC SIGNALING AND PLATELET ACTIVATION

Purinergic signaling is an important link among platelet activation, vascular thrombosis, and inflammation (Eltzschig et al., 2012). Mammalian cells contain high levels of ATP. Under pathologic conditions such as inflammation, there is an increased release of ATP. Extracellular adenosine is formed predominately from a series of enzymatic conversion from ATP, adenosine diphosphate (ADP), and AMP to adenosine (**Figure 2**). Adenosine signaling is terminated by uptaking adenosine from extracellular space to intracellular space and is then rapidly metabolized to inosine through adenosine deaminase (Eltzschig et al., 2006) or converted back to AMP through adenosine kinase (Morote-Garcia et al., 2008). Inhibition of adenosine kinase by cyclosporine resulting in increased levels of extracellular adenosine may contribute, at least in part, to the anti-inflammatory effects of cyclosporine (Spychala and Mitchell, 2002).

In human platelets, serotonin (5-HT) amplifies the aggregation induced by ADP (McBride et al., 1989; de Clerck, 1990), which is mediated by the 5-HT_2_ receptor complex. Thus, the magnitude of serotonin amplification of ADP-induced platelet aggregation and dense granule secretion (DGS) may provide us with an index to evaluate the platelet serotonin responsivity. In both normal control subjects and clinically stable patients with chronic SZ (with antipsychotic treatment), our laboratory demonstrated a robust increase of platelet aggregation in response to synergistic effects of ADP and 5-HT (Yao et al., 1996). Moreover, increases in 5-HT amplification were inversely correlated with the psychosis severity. The magnitude of 5-HT amplification, however, was not significantly different in those same patients after haloperidol withdrawal. Recently, we have further shown that FENNS patients have significantly lower 5-HT amplification than the normal control subjects (Reddy et al., 2007). The blunted platelet serotonergic responsivity may thus be associated with SZ *per se*, independent of drug effects. The magnitude of 5-HT amplification on ADP-induced platelet aggregation, however, can be augmented in SZ patients after eicosapentaenoic acid (EPA) supplementation (Yao et al., 2004b).

## DO PERIPHERAL INDICES OF METABOLIC DEFICITS ALSO REFLECT SIMILAR CHANGES IN THE BRAIN?

Whether peripheral indices of abnormal metabolites reflect similar changes in the brain and/or are related to presumed brain events are frequently raised by the reviewers in the grant applications and manuscript submissions. This issue has been vigorously debated because of examples in the literature, where peripheral measures either failed to adequately reflect central pathophysiology or did not serve as reliable biological markers. Therefore, in principle, the majority of research investigators believe that peripheral findings do not reflect the similar changes in the brain. However, in an editorial in Molecular Psychiatry, Wong and Licinio (2005) have eloquently stated that this belief has pervaded the field and has undermined our ability to confidently use the powerful tools of contemporary biology in order to dissect the biology of psychiatric disorders through investigation of peripheral markers, particularly those measured in peripheral blood.

Substantial evidence has been accumulated that reveals metabolic defects in both the peripheral and central tissue of patients with SZ (see reviews by Skosnik and Yao, 2003; Yao and van Kammen, 2004; Mahadik and Yao, 2006; Yao and Keshavan, 2011). Moreover, direct correlations between the peripheral (red blood cell, RBC) and central (31-phosphorus magnetic resonance spectroscopy, ^31^P MRS) phospholipids and polyunsaturated fatty acids (PUFAs) were shown in SZ patients (Richardson et al., 2001; Yao et al., 2002). Additionally, platelets and fibroblasts have been used as models for nerve cells in a variety of neuropsychiatric diseases (Farmer, 1980; Mahadik and Mukherjee, 1996). These findings support the notion that metabolic defects are present in both neural and extra-neural tissues, but the functional consequences may differ. For example, changes in peripheral metabolites may play a role in clinical presentation and outcome during the early course of SZ (Condray et al., 2011; Yao et al., 2012).

Moreover, there are several paradigmatic conditions such as Down syndrome, phenylketonuria, and various lipidoses (Scriver et al., 1989) where the metabolic abnormalities are expressed in both neural and peripheral tissues, but the functional consequences are most profound in the central nervous system (CNS). A recent review by Andrews and Neises (2012) also suggest that research examining the mechanism of how traumatic events are linked to peripheral blood mononuclear cell functions and biomarkers may offer improved diagnoses and treatments for post-traumatic stress disorder patients. This paradigm may also apply to SZ. A recent study by comparison of peripheral and central SZ biomarker profiles, Harris et al. (2012) have concluded that the systemic nature of SZ provides added validity of investigating blood-based biomarkers in SZ. If peripheral indices parallel central metabolic defects, and perhaps also neuromorphometric and/or neuroimaging findings, then there exists the possibility that alterations in peripheral indices on longitudinal follow-up (repeated measures) can usefully reflect central membrane function over the course of illness.

## CONCLUSION AND PERSPECTIVES

During the purine catabolism, there are three major purine bases and their corresponding ribonucleosides, which consist of adenine/adenosine, G/Gr, and Hx/inosine (**Figure 2**). As mentioned above, we have observed that a homeostatic imbalance of purine catabolism is present in FENNS. There are tightly correlated precursor and product relationships *within* purine pathways. Although some of these correlations persist across disease or medication status, others appear to be lost among FENNS (Yao et al., 2010b). Similar findings of lacking a control mechanism used by HC subjects were also demonstrated in the TRP pathway from these same FENNS patients (Yao et al., 2010a). When taken together, these observations suggest that a steady formation of the important antioxidant UA via purine catabolism is altered early in the course of illness.

Moreover, we have applied Kendall’s tau to assess correlations between purine metabolites and monoamine neurotransmitters with the Bonferroni corrections. Correlations between TYR, TRP, and some purines may originate in the diet or other common organism-wide influences, but some of these appear to be lost as these compounds undergo further transformations along their respective pathways. For both HC and patients, purine metabolites normally show significant cross-pathway correlation only with TYR, not with its metabolites, where correlations may be lost due to internal influences over neurotransmitter production. Furthermore, several purine metabolites (UA, Gr, or Xan) are each significantly correlated with TRP in all subjects. But purine correlations with 5-HIAA seem to be present only in HC subjects, not in FENNS at baseline or 4 weeks after antipsychotic treatment. Again, the loss of correlations in the pathway metabolite may be lost in patients due to illness-related, and also perhaps treatment influences, on 5-HIAA, since the TRP–5-HIAA correlation is appears weakened in patients (Yao et al., 2010a).

In conclusion, SZ is a heterogeneous disease with various abnormal metabolites involving multiple biochemical pathways. There is abundant evidence that alterations in key neurotransmitters can both be modified by and contribute to oxidative stress and membrane dysfunction (**Figure 1**), suggesting a link between these pathophysiological processes in SZ. GSH redox coupling, NO signaling, and purine catabolism are the key pathways involving the AODS. We have previously demonstrated a homeostatic imbalance of purine catabolism (Yao et al., 2010b) and blunted platelet serotonergic responsivity (Yao et al., 1996; Yao et al., 2004b; Reddy et al., 2007) in FENNS. In this “Hypothesis and Theory” paper, we propose that the altered purine metabolites have significantly impacts on not only within the purine catabolism but also across the TRP pathways involving the serotonin and KYN metabolism.

Firstly, several purine metabolites (UA, Gr, or Xan) are each significantly correlated with TRP in all subjects. However, purine correlations with 5-HIAA seem to be present only in HC subjects, not in FENNS at baseline or 4 weeks after antipsychotic treatment (**Table 2**). Conversion of serotonin to *N*-acetylserotonin by serotonin *N*-acetyltransferase may be upregulated in the same set of FENNS patients, possibly related to the observed alteration in TRP–5-HIAA correlation (Yao et al., 2010a). Lacking significant correlations between purine metabolites (UA and Xan) and 5-HIAA suggest that alterations in key serotonin pathways may both be modified by and contribute to oxidative stress via purine catabolism in FENNS.

Secondly, we have shown that a neurotoxic product of TRP metabolism, 3-OHKY, predicts severity of clinical symptoms during the early phase of illness and before exposure to antipsychotic drugs (Condray et al., 2011). Baseline level of 3-OHKY may also predict the degree of clinical improvement following brief treatment with antipsychotics. In the present paper, we have further demonstrated that levels of 3-OHKY were significantly correlated with levels of either Gr or UA in this same sample set. Considering the unique functional roles of UA (the end product of purine catabolism) as both antioxidant and pro-oxidant, the homeostatic balance of UA appears to play a vital role of regulatory functions in not only the AODS but also the KYN pathway. The KYN pathway that produces neurotoxic and neuroinhibitory compounds is regulated by the dopamine metabolites, VMA and HVA, which has been implicated in the pathogenic mechanisms underlying SZ.

Lastly, optimum levels of purine metabolites have been associated with the dynamics of clinical symptoms and therapeutic improvements, which may lead to discovery of novel targets for drug development. Interestingly, allopurinol, which is a structural isomer of Hx and is an inhibitor of the Xan oxidase, can improve SZ symptoms either when given alone or as add-on medication to haloperidol (Lara et al., 2001). Xan oxidase is responsible for the successive oxidation of Hx and Xan (), leading to the formation of UA (Pacher et al., 2006). Therefore, allopurinol may regulate levels of not only UA but also the extracellular adenosine (via feedback inhibition). Future investigations are required to establish therapeutic target for purinergic drugs in treatment of SZ patients.

## Conflict of Interest Statement

Dr. Rima Kaddurah–Daouk is a coinventor on a series of patents in the metabolomics field. [(1) One patent Issued (3/20/12) “Lipidomic approaches to determine drug response – phenotypes in cardiovascular disease” Patent # 8137977, expires 12/10/27. (2) One patent pending: “Lipidomic approaches for central nervous system disorders” Application # 12/091,213 filed 12/10/08, Publication # US 2009/0305323 12/10/09.] All other authors declare no conflict of interest.
